# Extracellular Histone-Induced Protein Kinase C Alpha Activation and Troponin Phosphorylation Is a Potential Mechanism of Cardiac Contractility Depression in Sepsis

**DOI:** 10.3390/ijms24043225

**Published:** 2023-02-06

**Authors:** Simon T. Abrams, Yasir Alhamdi, Min Zi, Fengmei Guo, Min Du, Guozheng Wang, Elizabeth J. Cartwright, Cheng-Hock Toh

**Affiliations:** 1 Department of Clinical Infection Microbiology and Immunology, University of Liverpool, Liverpool L69 7BE, UK; 2 Coagulation Department, Liverpool University Hospitals NHS Foundation Trust, Liverpool L7 8XP, UK; 3 Sheffield Teaching Hospital NHS Foundation Trust, Sheffield S5 7AU, UK; 4 Institute of Cardiovascular Sciences, Centre for Cardiac Research, University of Manchester, Manchester M13 9PT, UK; 5 The Medical School, Southeast University, Nanjing 210009, China; 6 Roald Dahl Haemostasis & Thrombosis Centre, Royal Liverpool University Hospital, Liverpool L7 8XP, UK

**Keywords:** sepsis, cardiac contractility, protein kinase C (PKC), troponin, phosphorylation

## Abstract

Reduction in cardiac contractility is common in severe sepsis. However, the pathological mechanism is still not fully understood. Recently it has been found that circulating histones released after extensive immune cell death play important roles in multiple organ injury and disfunction, particularly in cardiomyocyte injury and contractility reduction. How extracellular histones cause cardiac contractility depression is still not fully clear. In this work, using cultured cardiomyocytes and a histone infusion mouse model, we demonstrate that clinically relevant histone concentrations cause significant increases in intracellular calcium concentrations with subsequent activation and enriched localization of calcium-dependent protein kinase C (PKC) α and βII into the myofilament fraction of cardiomyocytes in vitro and in vivo. Furthermore, histones induced dose-dependent phosphorylation of cardiac troponin I (cTnI) at the PKC-regulated phosphorylation residues (S43 and T144) in cultured cardiomyocytes, which was also confirmed in murine cardiomyocytes following intravenous histone injection. Specific inhibitors against PKCα and PKCβII revealed that histone-induced cTnI phosphorylation was mainly mediated by PKCα activation, but not PKCβII. Blocking PKCα also significantly abrogated histone-induced deterioration in peak shortening, duration and the velocity of shortening, and re-lengthening of cardiomyocyte contractility. These in vitro and in vivo findings collectively indicate a potential mechanism of histone-induced cardiomyocyte dysfunction driven by PKCα activation with subsequent enhanced phosphorylation of cTnI. These findings also indicate a potential mechanism of clinical cardiac dysfunction in sepsis and other critical illnesses with high levels of circulating histones, which holds the potential translational benefit to these patients by targeting circulating histones and downstream pathways.

## 1. Introduction

Sepsis is still the leading cause of death in most intensive care units (ICU) with an unacceptably high mortality rate (10–20%) due to multiple organ dysfunction [1,2]. Reduction in cardiac contractility is common in severe sepsis [3,4]. However, the pathological mechanism is still not fully understood [5]. Certain pathogen-associated molecular patterns (PAMPs), such as pneumolysin, lipopolysaccharide (LPS) [6,7,8]; damage-associated molecular patterns (DAMPs), such as histones, HMGB1 [7,9,10]; cytokines, such as tumour necrosis factor-alpha (TNF-α), interleukin-1-beta (IL-1β) and IL-6 [3,11] may lead to direct myocardial suppression. Other factors, such as nitric oxide (NO), reactive oxygen species (ROS), low levels of high-density lipoprotein (HDL) and metabolic dysfunction of mitochondria may also be involved [5,12,13,14]. Recently the roles of circulating histones released after extensive immune cell death in multiple organ injury and disfunction, particularly in cardiomyocyte injury, have drawn great attention [10,15,16,17].

Histones are intra-nuclear proteins that play an essential role in the assembly of chromatin and the regulation of gene expression [18,19]. However, the seminal work by Xu et al. demonstrated that histones released extracellularly upon extensive cell injury can be mediators of death in sepsis [20]. Several reports have since confirmed that high circulating histone levels contribute to organ injury [16] and death in experimental animals as well as in patients with sepsis [10,21], trauma [22,23], pancreatitis [24] and other critical illnesses [25]. In particular, recent studies have demonstrated that circulating histones are novel mediators of cardiac injury and dysfunction in septic animals and patients [10,17]. High circulating histone levels in septic patients associate significantly and robustly predict the development of new-onset left ventricular (LV) dysfunction and arrhythmias, with a strong correlation with well-recognized circulating biomarkers of cardiac injury (i.e., cardiac troponins) [10]. These cardiac complications were reproducible in septic mouse models [10,17] as well as in histone infusion mouse models [26] and that anti-histone antibody treatment could significantly abrogate these cardiac events [10,17,26]. These advances provide strong evidence for the cardio-toxic profile of histones and their likely role in the pathogenesis of septic cardiomyopathy [27].

The cardiotoxicity of histones was related to injury at the cardiomyocyte level, primarily through increasing intracellular calcium concentrations ([Ca^2+^]_i_) to pathological levels [17,26]. This was mainly mediated by histone binding to cardiomyocyte membranes causing a profound Ca^2+^ influx and overload [26]. Ca^2+^ is the principal regulator of cardiomyocyte contractility [28,29] and disturbed calcium handling in cardiomyocytes is well recognized as a central player in cardiac mechanical and electrophysiological disturbances [29,30,31]. In cardiomyocytes, Ca^2+^ interacts with the cardiac troponin complex (particularly with cardiac troponin C “cTnC”), which acts as a Ca^2+^ sensor that regulates the extent and velocity of cross-bridge machinery between actin and myosin that triggers contraction and relaxation in cardiomyocytes [32,33]. As such, it is now well understood that cardiomyocyte contractility can be modulated in a physiological and/or pathological setting by (1) the amplitude and duration of the Ca^2+^ transient (difference between the systolic and diastolic levels of [Ca^2+^]_i_) and (2) the sensitivity or the responsiveness of cardiac myofilaments (particularly the troponin complex) to Ca^2+^ [33,34,35,36].

Cardiac troponins regulate cardiomyocyte contraction via the calcium-mediated interaction between actin and myosin. Regulated phosphorylation of troponin I determines troponin positioning for optimal regulation of cardiac muscle contraction [37,38]. Protein kinase A (PKA), protein kinase C (PKC) and their isoforms are the most important kinases related to troponin phosphorylation [34,35] which alter cardiac myofilament properties and ultimately affect inotropy (contraction) and lusitropy (relaxation) of cardiomyocytes [34,35,38,39]. Abnormally increased cTnI phosphorylation by PKCs has been shown to be detrimental to cardiomyocyte contractility in vitro and in vivo [8,40,41,42,43] with such changes also reported in failing human hearts [44].

In this study, we aimed to investigate the effect of extracellular histones on cTnI phosphorylation status and the functional consequences on cardiomyocyte contractility. Our results show that extracellular histones increase cTnI phosphorylation in a dose-dependent manner, mediated by enhanced PKCα activation and association with cardiomyocyte myofilaments. Inhibiting PKCα significantly abrogated cTnI phosphorylation and resulted in significant improvement in different mechanical properties of cardiomyocyte contractility, including peak shortening (i.e., peak contraction), duration of systolic and diastolic cycles and velocity of shortening (contraction) and re-lengthening (relaxation).

## 2. Results

### 2.1. Histones Disturb Intracellular Calcium Dynamics

**Figure 1:**
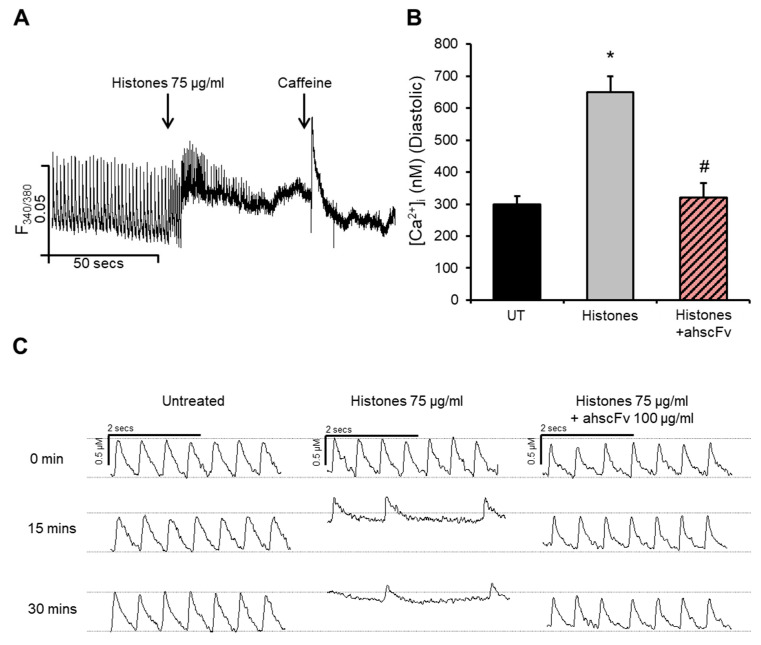
Histones increase intracellular calcium concentrations and disturb calcium dynamics in cardiomyocytes. (**A**) Changes in intracellular calcium concentrations [Ca^2+^]_i_ in HL-1 cardiomyocytes following histone (75 µg/mL) treatment as recorded using florescence photometry with Fura-2AM as a Ca^2+^ indicator. Upper edge of the trace represents systolic [Ca^2+^]_i_ and bottom edge represents diastolic [Ca^2+^]_i_. Caffeine (10 mM) was used as a positive inducer of Ca^2+^ release from sarcoplasmic reticulum stores. *n* = 4. (**B**) Diastolic levels of [Ca^2+^]_i_ recorded 1 min after treatment with culture medium (untreated “UT”), histones 75 µg/mL (histones) and histones 75 µg/mL + anti-histone antibody 100 µg/mL (histones + ahscFv). *n* = 4. * *p* < 0.05 as compared to UT, # *p* < 0.05 as compared to histone treatment. (**C**) Representative traces of Ca^2+^ waves over time in untreated cells and in cardiomyocytes treated with histones 75 µg/mL ± ahscFv 100 µg/mL. Solid horizontal bar represents a time duration of 2 s. Dotted horizontal bars represent systolic (upper line) and diastolic (bottom line) [Ca^2+^]_i_ in baseline untreated cardiomyocytes. *n* = 9.

We have previously demonstrated that histones induce substantial increases in [Ca^2+^]_i_ in cardiomyocytes through Ca^2+^ influx [26]. HL-1 cardiomyocyte exposure to histones at 75 µg/mL, a concentration previously shown to strongly correlate with and predict new-onset LV dysfunction and arrhythmias in septic patients [10], induced significant increases in [Ca^2+^]_i_ (Figure 1A). Diastolic (resting) [Ca^2+^]_i_ more than doubled (increased from 300 ± 24 nM to 650 ± 50 nM) following histone exposure, an effect that was significantly abrogated by anti-histone (ahscFv) treatment (Figure 1A,B). These results are in agreement with our recent report [26]. Here, we further extend these results by showing that in addition to increasing [Ca^2+^]_i_, histones caused substantial deterioration in Ca^2+^ cycling with time-dependent reductions in the frequency of Ca^2+^ waves from 4–5 waves/s to approximately 1–2 waves/s after 15–30 min of treatment (Figure 1C), which was also associated with irregularity in the time duration between individual Ca^2+^ cycles (Figure 1C). These effects were significantly attenuated by ahscFv treatment (Figure 1C) whose specific histone-binding properties have been previously demonstrated in vitro and in vivo [10,22,26].

### 2.2. Histones Induce Ca^2+^-Dependent PKC Isoform Activation in Cardiomyocytes

**Figure 2:**
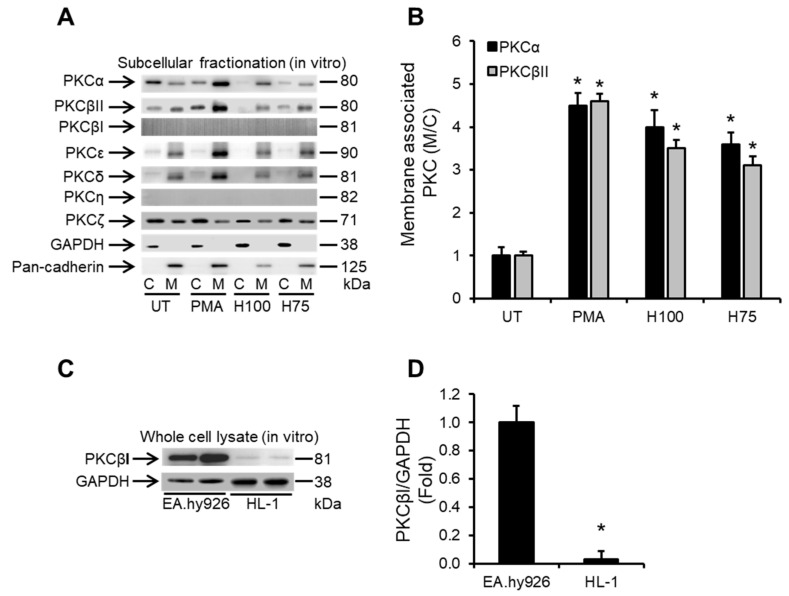
Histones induce the translocation of PKCα and PKCβII from the cytosolic to membrane fractions of cardiomyocytes. (**A**) Western blots illustrating the cytosolic “C” to membrane “M” distribution of protein kinase C (PKC) α, PKCβII, PKCβI, PKCε, PKCδ, PKCη and PKCζ in HL-1 cardiomyocytes in untreated conditions (UT) or following 30 min of treatment with histones at 75 and 100 µg/mL (H75 and H100, respectively). Phorbol myristate acetate (PMA) was used as a positive inducer of PKCα and PKCβII activation (i.e., translocation from C to M fractions of cardiomyocytes). GAPDH and pan-cadherin were used as markers of C and M fractions, respectively. *n* = 4. (**B**) Band-quantification histogram illustrating the cytosolic “C” to membrane “M” distribution of PKCα and PKCβII. *n* = 4. * *p* < 0.05 as compared to UT. (**C**) Western blots illustrating the level of expression of PKCβI in whole cell lysates from HL-1 cardiomyocytes and EA.hy926 endothelial cells. GAPDH was used as a loading control. (**D**) PKCβI/GAPDH ratios from band-quantification, *n* = 3. * *p* < 0.05 as compared to EA.hy926.

PKC isoforms and particularly classical Ca^2+^-dependent PKCs, e.g., PKCα and β, have well-documented roles in cardiac pathologies and in suppressing cardiac contractility [8,41,45]. This is mainly through altering the phosphorylation status of thin myofilaments [8,34,35,41]. Since histones induce profound Ca^2+^ overload in cardiomyocytes, we investigated whether this could trigger the activation of PKC signalling in cardiomyocytes. We found that extracellular histones (75 and 100 µg/mL) strongly induced translocation of both PKCα and PKCβII from the cytosol to the membrane compartment of HL-1 cardiomyocytes (Figure 2A,B), which is a hallmark of PKC activation [46,47]. Interestingly, histone treatment only induced translocation of the Ca^2+^-dependent PKC isoforms: PKCα and PKCβII after 30 min of treatment (Figure 2A,B), whereas all the Ca^2+^-insensitive PKC isoforms, including the novel PKCs (PKCε, PKCδ, PKCη) and atypical PKCs (PKCζ), were unaffected by histone treatment of HL-1 cardiomyocytes (Figure 2A). The Ca^2+^-sensitive PKCβI and Ca^2+^-insensitive PKCη were not detectable in the subcellular cytoplasmic or membrane fractions of HL-1 cardiomyocytes (Figure 2A) and whole cell lysate preparation revealed that these PKC isoforms were present in very small amounts in HL-1 cardiomyocytes (Figure 2C,D). Phorbol-12-myristate-13-acetate (PMA) was used as a positive control for Ca^2+^-dependent PKC activation [8] (Figure 2A,B).

### 2.3. Histones Enhance the Association of PKCα and PKCβII with Cardiac Myofilaments and Induce cTnI Phosphorylation

**Figure 3:**
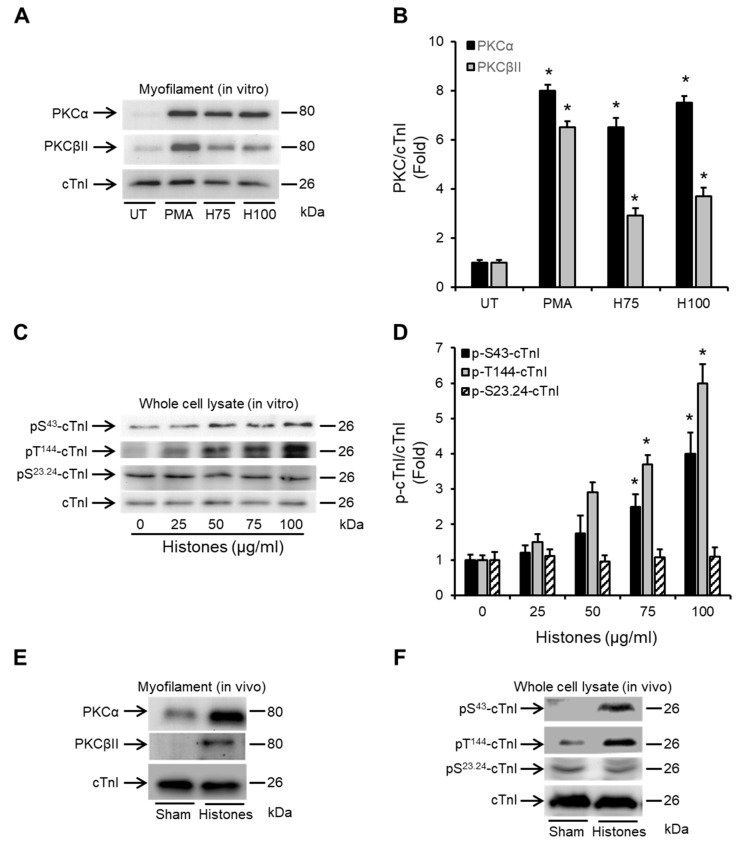
Histones increase the association of PKCα and PKCβII to the myofilament fraction of cardiomyocytes. (**A**,**B**) Western blots of the myofilament (triton-insoluble) fraction of HL-1 cardiomyocytes (**A**) and band-quantification histogram (**B**) illustrating the levels of myofilament-associated PKCα and PKCβII in untreated cells (UT) or 30 min after treatment with histones at 75 and 100 µg/mL (H75 and H100, respectively). Phorbol myristate acetate (PMA) was used as a positive inducer of PKCα and PKCβII activation. Cardiac troponin I (cTnI) was used as a marker of the myofilament fraction of HL-1 cardiomyocytes and a loading control. *n* = 4. * *p* < 0.05 as compared to UT. (**C**,**D**) Western blots (**C**) and band-quantification histogram (**D**) illustrating the phosphorylation status of cardiac troponin I (cTnI) at the PKC-regulated residues (S43 and T144) and PKA-regulated phosphorylation residues (S23.S24) 30 min following HL-1 cardiomyocytes treatment with different doses of histones. Specific antibodies against phosphorylated cTnI at S43 (pS^43^-cTnI), T144 (pT^144^-cTnI) and S23.24 (pS^23.24^-cTnI) were used. cTnI (total) was used as a loading/endogenous control. *n* = 4. * *p* < 0.05 as compared to 0 µg/mL histones. (**E**) Western blots of the myofilament (triton-insoluble) fraction of murine cardiomyocytes illustrating the levels of myofilament-associated PKCα and PKCβII in mice infused with histones at 30 mg/kg (histones) or an equal volume of saline (sham). Murine hearts were harvested 60 min after histone or saline infusion. Cardiac troponin I (cTnI) was used as a marker of the myofilament fraction and a loading control. *n* = 4. (**F**) Western blots illustrating the phosphorylation status of cardiac troponin I (cTnI) at the PKC-regulated residues (S43 and T144) and PKA-regulated phosphorylation residues (S23.S24) 60 min following intravenously infusing mice with histones (30 mg/kg) or an equal volume of normal saline. Specific antibodies against phosphorylated cTnI at S43 (pS^43^-cTnI), T144 (pT^144^-cTnI) S23.24 (pS^23.24^-cTnI) were used. cTnI (total) was used as a loading/endogenous control. *n* = 4.

While the translocation of PKCα and PKCβII from the cytosol to the membrane fraction of cardiomyocytes suggest activation of these PKC isoforms in cardiomyocytes following histone exposure, functional consequences are typically associated with enhanced localization with the myofilament fraction of cardiomyocytes where they can directly target the different PKC-regulated sites on cTnI [8,34,35,41]. Histones at 75 and 100 μg/mL increased the myofilament association of PKCα by 6.39 ± 0.41 fold and 7.53 ± 0.27 fold, respectively and of PKCβII by 2.9 ± 0.31 fold and 3.7 ± 0.36 fold, respectively, compared to untreated HL-1 cardiomyocytes, suggesting a stronger effect on PKCα (Figure 3A,B).

Specifically, there was histone-induced dose-dependent phosphorylation of cTnI at the PKC-regulated residues (S43 and T144) (Figure 3C). Phosphorylation levels were significantly higher when HL-1 cardiomyocytes were exposed to 75 and 100 µg/mL histones compared to untreated cells (Figure 3D). Histone treatment did not influence the phosphorylation of cTnI at the protein kinase A (PKA)-regulated residue (S23.S24) (Figure 3C,D). Importantly, intravenous injection of histones into mice at a concentration of 30 mg/kg (resulting in circulating histone concentrations of approximately 65.5 ± 13.4 µg/mL) [26], that causes direct LV dysfunction [26], resulted in enhanced PKCα and PKCβII association with cardiac myofilaments (Figure 3E) and also substantial induced cTnI phosphorylation at the PKC-regulated sites (S43 and T144) in murine cardiomyocytes (Figure 3F). Histone treatment did not influence the phosphorylation of cTnI at the protein kinase A (PKA)-regulated residue (S23.S24) in vivo (Figure 3F).

### 2.4. Histone-Induced cTnI Phosphorylation Is Mediated by PKCα and Contributes to Depressed Cardiomyocyte Contractility

**Figure 4:**
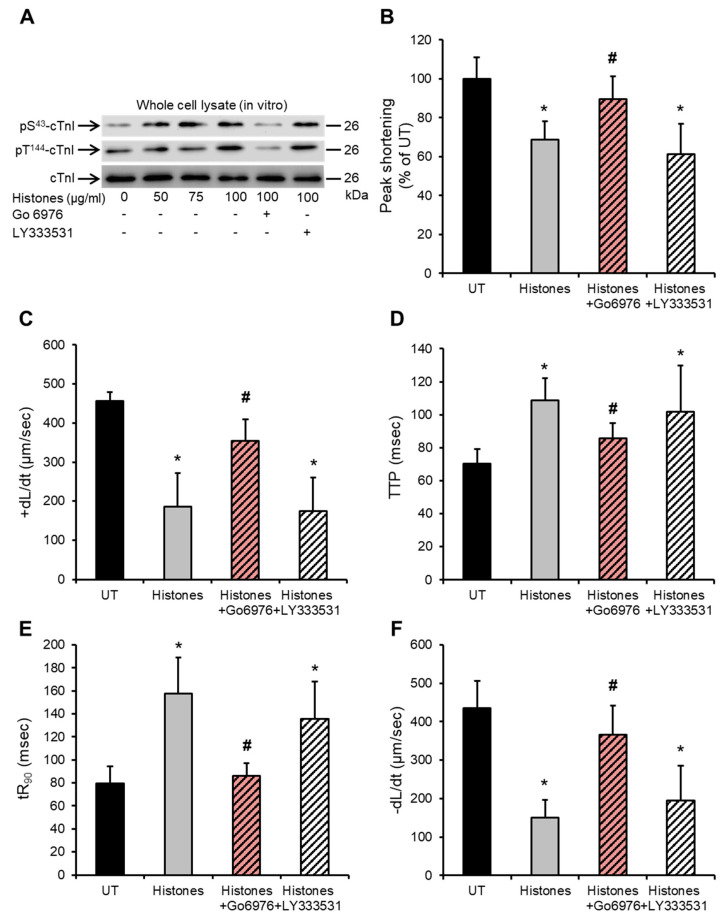
PKCα inhibition abrogates histone-induced cTnI phosphorylation and improves cardiomyocyte contractility. (**A**) Representative Western blots illustrating the effects of a PKCα inhibitor (Go6976 5 nM) and PKCβII inhibitor (LY333531 10 nM) on histone-induced cardiac troponin I (cTnI) phosphorylation at PKC-regulated phosphorylation residues (S43 and T144) in HL-1 cardiomyocytes. Specific antibodies against phosphorylated cTnI at S43 (pS^43^-cTnI) and T144 (pT^144^-cTnI) were used. cTnI (total) was used as a loading/endogenous control. *n* = 4. (**B**–**F**) Effects of histones (75 µg/mL) ± PKCα inhibitor (Go6976 5 nM) or PKCβII inhibitor (LY333531 10 nM) on peak shortening (untreated “UT” cardiomyocyte level set to 100%), maximum velocity of shortening (+dL/dt), time to peak shortening (TTP), time to 90% re-lengthening (tR_90_) and maximum velocity of re-lengthening (−dL/dt). *n* = 9. * *p* < 0.05 as compared to UT, # *p* < 0.05 as compared to histone treatment.

Since histones induced enhanced localization of Ca^2+^-dependent PKCα and PKCβII into the cardiac myofilaments both in vitro and in vivo, we further explored which PKC isoforms might be responsible for the observed histone-induced cTnI phosphorylation at the PKC-regulated residues (S43 and T144). Using a specific PKCα inhibitor Go6976 (5 nM) (IC50 for PKCα is 2.3–5 nM) and a specific PKCβII inhibitor 10 nM LY333531 (IC50 for PKCβII is 10 nM) [8,48], we found that blocking PKCα significantly attenuated the histone-induced enhanced phosphorylation of cTnI at both PKC-regulated sites (S43 and T144) (Figure 4A) in HL-1 cardiomyocytes, whereas the PKCβII inhibitor had no detectable effect on cTnI phosphorylation status (Figure 4A).

Since histones at 75 µg/mL induce substantial deterioration in various mechanical properties of cardiomyocytes contractility [26], functional consequences from inhibiting PKCα and PKCβII following histone treatment of HL-1 cardiomyocytes were then investigated. Blocking PKCα using Go6976 (5 nM) prior to treating cardiomyocytes with histones (75 μg/mL) resulted in significant improvements in peak shortening (untreated “UT” set as 100%, histones 68.7 ± 9.2%, histones + Go6976 89.6 ± 11%) (Figure 4B), maximum velocity of shortening (+dL/dt) (UT 455 ± 23 µm/s, histones 186 ± 84 μm/s, histones + Go6976 354 ± 55 μm/s) (Figure 4C), time to peak (TTP) (systolic duration) (UT 70 ± 8 ms, histones 108 ± 13 ms, histones + Go6976 85 ± 9ms) (Figure 4D), time to 90%-re-lengthening (tR_90_) (diastolic duration) (UT 79 ± 15 ms, histones 157 ± 31 ms, histones + Go6976 86 ± 10 ms) (Figure 4E) and in maximum velocity of re-lengthening (−dL/dt) (UT 435 ± 70 μm/s, histones 150 ± 46 μm/s, histones + Go6976 366 ± 74 μm/s) (Figure 4F). On the other hand, blocking PKCβII using LY333531 (10 nM) prior to histone treatment showed no obvious protective effects on peak shortening (histones + LY333531 61 ± 15%) (Figure 4B), +dL/dt (histones + LY333531 175 ± 85 μm/s) (Figure 4C), TTP (histones + LY333531 101 ± 72 ms) (Figure 4D), tR_90_ (histones + LY333531 103 ± 32 ms) (Figure 4E) or −dL/dt (histones + LY333531 195 ± 90 μm/s) (Figure 4F).

### 2.5. Anti-Histone Antibody Abrogates Histone-Induced Activation of PKCα-cTnI Pathway to Rescue Cardiomyocyte Contractility

**Figure 5:**
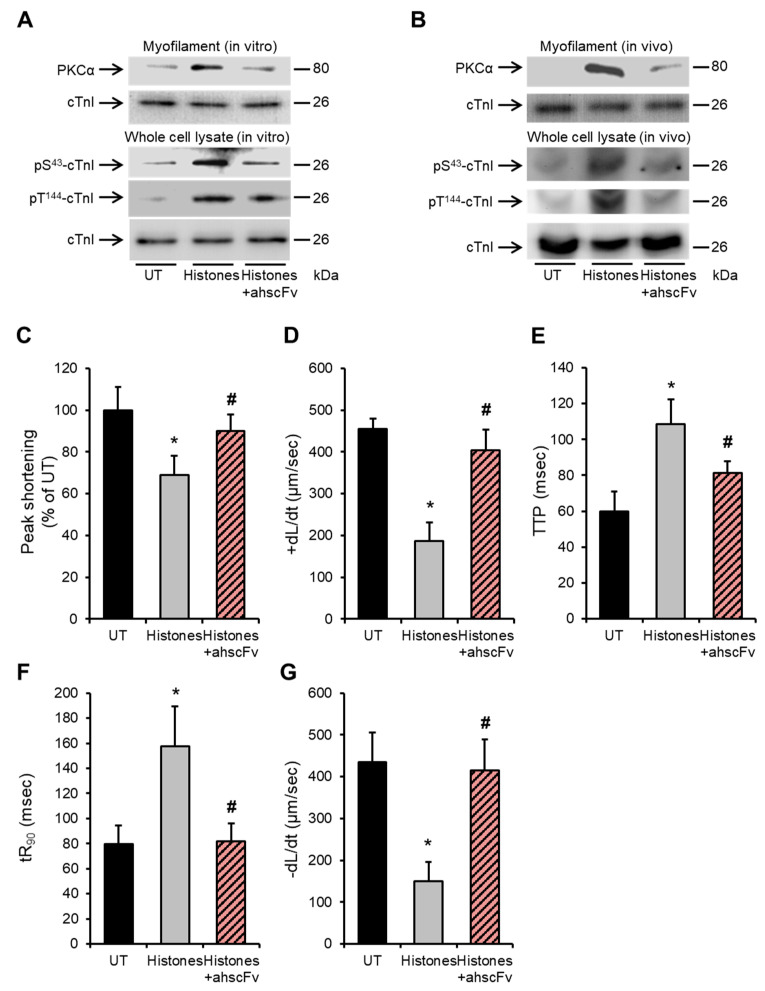
Anti-histone antibody abrogates histone-induced cTnI phosphorylation and improves cardiomyocyte contractility. (**A**) Upper panel: representative Western blots of the myofilament (triton-insoluble) fraction of HL-1 cardiomyocytes illustrating the levels of myofilament-associated PKCα in untreated cells (UT) or 30 min after treatment with histones at 75 µg/mL ± 100 µg/mL anti-histone antibody (ahscFv). Cardiac troponin I (cTnI) was used as a marker of the myofilament fraction of HL-1 cardiomyocytes and a loading control. *n* = 4. Bottom panel: representative Western blots illustrating the effects of the anti-histone antibody (100 µg/mL) on histone-induced cTnI phosphorylation at the PKC-regulated phosphorylation residues (S43 and T144) in HL-1 cardiomyocytes. Specific antibodies against phosphorylated cTnI at S43 (pS^43^-cTnI) and T144 (pT^144^-cTnI) were used. cTnI (total) was used as a loading/endogenous control. *n* = 4. (**B**) Upper panel: Representative Western blots of the myofilament (triton-insoluble) fraction of murine cardiomyocytes illustrating the levels of myofilament-associated PKCα in saline-infused mice (Sham) or 60 min after histones intravenous (i.v.) infusion at 30 mg/kg ± 10 mg/kg anti-histone antibody (ahscFv). Cardiac troponin I (cTnI) was used as a marker of the myofilament fraction of HL-1 cardiomyocytes and a loading control. *n* = 4. Lower panel: Representative Western blots illustrating the effects of anti-histone antibody on histone-induced cTnI phosphorylation at the PKC-regulated phosphorylation residues (S45 and T144) in murine cardiomyocytes. Specific antibodies against phosphorylated cTnI at S45 (pS45-cTnI) and T144 (pT144-cTnI) were used. cTnI (total) was used as a loading/endogenous control. *n* = 4. (**C**–**G**) Effects of histones (75 µg/mL) ± 100 µg/mL anti-histone antibody (ahscFv) on peak shortening (untreated “UT” cardiomyocyte level set to 100%), maximum velocity of shortening (+dL/dt), time to peak shortening (TTP), time to 90% re-lengthening (tR_90_) and maximum velocity of re-lengthening (−dL/dt). *n* = 9. * *p* < 0.05 as compared to UT, # *p* < 0.05 as compared to histone treatment.

We have previously demonstrated the protective effect of the anti-histone antibody (ahscFv) against histone-induced cardiac injury and dysfunction in septic mice [10] and in a histone-infusion mouse model [26]. However, its protective effect against detrimental signalling pathways in cardiomyocytes has not been previously tested. Figure 5A shows that ahscFv (100 µg/mL) significantly attenuated PKCα localization to cardiomyocyte myofilaments (upper panel) and significantly abrogated cTnI phosphorylation at the S43 and T144 phosphorylation residues (bottom panel) following 75 µg/mL histone treatment. Likewise, anti-histone antibody infusion into mice, abrogated histone-induced PKCα increased association with LV myofilaments and cTnI phosphorylation at the PKC-regulated residues (p-T144 and p-S43) (Figure 5B).

Furthermore, ahscFv treatment significantly abrogated the histone-induced deterioration in peak shortening (untreated “UT” set as 100%, histones 68.7 ± 9.2%, histones + ahscFv 90 ± 7%) (Figure 5C), +dL/dt (UT 455 ± 23 µm/s, histones 186 ± 84 μm/s, histones + ahscFv 404 ± 48 μm/s) (Figure 5D), TTP (systolic duration) (UT 70 ± 8 ms, histones 108 ± 13 ms, histones + ahscFv 81 ± 6 ms) (Figure 5E), tR_90_ (diastolic duration) (UT 79 ± 15 ms, histones 157 ± 31 ms, histones + ahscFv 82 ± 14 ms) (Figure 5F), and −dL/dt (UT 435 ± 70 μm/s, histones 150 ± 46 μm/s, histones + ahscFv 415 ± 78 μm/s) (Figure 5G).

## 3. Discussion

Circulating histones released following extensive cellular injury in patients and mouse models of sepsis were recently reported to be novel mediators of cardiac injury, new-onset LV dysfunction and arrhythmia [10]. Using a histone infusion mouse model, the dose-dependent cardio-toxic profile of circulating histones on LV and RV functions at clinically relevant histone concentrations was systematically elucidated [26]. These reports and the work of others have established that histone-induced cardiomyocyte contractile dysfunction is mediated by pathological Ca^2+^ overload with our work illustrating its mediation mainly through histone-induced Ca^2+^ influx into cardiomyocytes following binding to cardiomyocyte membranes [26]. This report extends these findings and demonstrates that histones can induce activation of the “classical” Ca^2+^-dependent PKC isoforms. These are mainly PKCα and PKCβII in cultured cardiomyocytes in vitro and in vivo in murine cardiomyocytes following histone infusion. This activation is most likely attributed to the substantial increase in the intracellular Ca^2+^ concentration following histone treatment. This line of reasoning is further strengthened by the finding that histones had no effect on the activation of Ca^2+^-insensitive PKC isoforms that belong to the “novel” and “atypical” classes of PKCs, which are usually activated by agents that result in diacylglycerol accumulation (novel PKCs) or from direct protein–protein interactions (atypical PKCs) [46]. The exclusive activation of Ca^2+^-dependent PKCs in cardiomyocytes in vitro and in vivo following histones exposure therefore emphasizes the key role of Ca^2+^ overload in histone-induced cardiomyocyte dysfunction.

The role of PKC isoforms in regulating cardiomyocyte contractility has been extensively studied in various scenarios and pathological conditions [39,49]. Although some controversies remain with regard to the direct effects of the different PKC isoforms on cardiac function [50,51,52], there are also well-established facts. Among these are the adverse consequences of PKCα activation on cardiomyocyte contractility and its role in the development of various myocardial disorders [8,31,41,51], which has led to PKCα being proposed as a novel therapeutic target for heart failure [45,53]. However, the role of PKCβII in regulating cardiac function in physiological and pathological conditions is less obvious and more controversial [50,51,52]. Our results are in line with these reports. Although histones activated both PKCα and PKCβII and dramatically increased their association with cardiac myofilaments, PKCα had a much more dominant role in regulating cardiomyocyte contractility since inhibiting this isoform largely relieved the histone-induced disturbances on different mechanical properties of contraction (i.e., force, velocity and length of systolic and diastolic durations) whereas blocking PKCβII did not convey any effects.

Furthermore, our data suggest that PKCα contributes to contractility suppression, following cardiomyocyte exposure to histones, by directly phosphorylating the PKC-dependent sites on cTnI (S43 and T144). The PKCα inhibitor (Go 6976) blocked the histone-mediated phosphorylation of S43 and T144, whereas PKCβII inhibition showed no similar molecular effects on cTnI phosphorylation, to support the hypothesis that histones contribute to the suppression of cardiomyocyte contractility through activation of the PKCα-cTnI axis. This observation is supported by numerous studies in various settings reporting that increased cTnI phosphorylation contributes to reduced responsiveness of cardiac myofilaments to Ca^2+^ and ultimately depressed contractility [8,40,41,42,44,50]. Importantly, histone-induced PKCα activation and cTnI phosphorylation at S43 and T144 sites was also observed following histone infusion into mice. This further highlights the relevance of this pathway in vivo, as has been recently shown by a pore-forming bacterial toxin causing cardiac injury when infused into mice [8].

Certain points relating to the effects of troponin phosphorylation on cardiomyocyte contraction remain controversial. It seems that the balance between complex rigidity/plasticity and calcium sensitivity as well as secondary phosphorylation may be major issues in determining the overall effects [54]. It is reported that in cTnI, S43/45 communicates with S23/24 and T144 [55]. S23/24 phosphorylation, predominantly by PKA, shift myofilament Ca^2+^ sensitivity to contribute to accelerated relaxation [56,57]. S43/45 and T144 are predominantly phosphorylated by PKCα. S43/45 phosphorylation acts as a brake to reduce the contractility and re-lengthening [58]. However, the effect of T144 phosphorylation is not well defined and remains controversial [54]. Our findings demonstrate that S43 and T144 were phosphorylated simultaneously and caused reduced contractility, similar to other reports that used cTnI-S43Asp, or cTnI_PKC-P_ to mimic phosphorylation [59,60]. How the multiple phosphorylation sites on troponins work together still requires extensive investigation both in vitro and in vivo to clarify their dynamic roles.

Work presented here also suggests that there is considerable translational potential for the development of anti-histone antibodies in combating histone-induced cardiomyocyte dysfunction. Such antibodies have been previously demonstrated to significantly abrogate the histone-induced detrimental effects on LV function in septic mice [10,17], LV and RV dysfunction in a histone infusion mouse model [26] and also prevent histone-specific cardiomyocyte death following incubation with septic patients’ plasma with high circulating histones [10]. Our findings in this report further extend these results by illustrating that anti-histone antibodies can attenuate PKCα activation and subsequent cTnI phosphorylation in cardiomyocytes in vitro and in vivo.

Although the in vitro signalling experiments were performed with HL-1 cells and not primary adult cardiomyocytes, previous work has demonstrated that HL-1 cells are a suitable model for cardiomyocyte signalling (including for PKC signalling and cTnI phosphorylation pathways) [8,61] as well as to test various cardio-toxic agents and conditions [62,63,64], with comparable results to those obtained in the mouse heart. This work further confirms that the results obtained in HL-1 cardiomyocytes are consistent with those observed in mouse LVs.

While previous reports have already demonstrated that increased PKCα activation and cTnI phosphorylation is a well-recognized feature of cardiac dysfunction in animal models and patients with depressed cardiac function [31,40,41,42,44,45,51,53], this new finding suggests that future studies may be tailored to explore the association between circulating histone levels and the activation of the PKCα-cTnI pathway with implications in cardiac function. This is particularly important in patients with sepsis since these pathways are known to be activated [40,42] and recent reports have established the relevance of circulating histones to the cardiac dysfunction of sepsis. It must also be emphasized that since histones induce Ca^2+^ overload in cardiomyocytes, it is unlikely that PKCα activation and cTnI phosphorylation are the only detrimental pathways that are activated in cardiomyocytes following exposure to high levels of circulating histones in sepsis. Other kinases, such as PKA and PKD, are also involved in the regulation of cardiac contractility [37,38,65] by finely adjusting troponin–actin–myosin complexes. Further studies to explore the detailed effects of extracellular histones on other components of cardiac myofilaments and other calcium-sensitive pathways in cardiomyocytes are warranted.

## 4. Materials and Methods

### 4.1. HL-1 Cardiomyocyte Culture

HL-1 cardiomyocytes were a kind gift from Dr. W. Claycomb (Louisiana State University, Baton Rouge, LA, USA). These cells which have typical features of adult cardiomyocytes and maintain spontaneous contractility, were cultured as previously described [8,10,26] in Claycomb medium (Sigma-Aldrich, Gillingham, UK) supplemented with 10% foetal bovine serum (Sigma-Aldrich, Gillingham, UK), 2 mM L-glutamine (Gibco, Paisley, UK), 100 U/mL penicillin–streptomycin (Gibco, Paisley, UK) and 100 µM norepinephrine (Sigma-Aldrich, Gillingham, UK). When fully confluent, cells showed spontaneous contraction at a rate of 5–6 Hz (5–6 beats/s) at 37 °C and 5% CO_2_. Medium was changed every 24 h and cells were passaged only when they reached full confluency (twice a week) as evident by the presence of clusters of spontaneously contracting cells (≥70% of cells). All flasks, tissue culture dishes and well-plates were pre-coated with 5 µg/mL fibronectin and 0.02% gelatine for at least 60 min before being seeded with HL-1 cardiomyocytes.

### 4.2. Animal Experiments

All mice experiments were performed in accordance with the UK Home Office and institutional guidelines (Project license PPL 40/3625). Twelve-week-old male C57BL/6N mice (body weight, 24–27 g) were purchased from Charles River Laboratories (Oxford, UK) and kept in a pathogen-free facility at the University of Manchester. Since we previously demonstrated that different sources of histones (calf thymus, recombinant, isolated human or murine histones) express similar toxicity, calf thymus histones (endotoxin-free) (Roche, Welwyn Garden City, UK) were used for animal experiments, as described [26]. In brief, mice were anaesthetized with avertin (200 mg/kg) and heart rates were maintained at around 450 beats/min. Histones at 30 mg/kg dissolved in normal saline (3 μg/μL concentration and infusion at 40 μL/min) were infused through the left subclavian vein, with the same saline volume infused in sham mice (control). In some experiments, anti-histone single chain variable fragment (ahscFv) antibody was infused into mice at 10 mg/kg through a different tail vein after completion of histone infusion, as previously described [26]. Mice were euthanized (1 h after the infusion) by neck dislocation without recovery from anaesthesia and left ventricles (LVs) were collected. LVs were divided into three portions, one to prepare whole cardiomyocyte lysates and the other two for isolating sub-cellular fractions (cytoplasmic, membrane and myofilament fractions), as detailed below.

### 4.3. Ca^2+^ Flux Measurement in HL-1 Cardiomyocytes

Ca^2+^ flux experiments in HL-1 cardiomyocytes were performed as previously described [8,26]. Briefly, 10^6^ cardiomyocytes were seeded into 35 mm glass-bottom tissue culture dishes (Corning, Flintshire, UK) and cultured as described above. When confluent and spontaneously contracting (2–3 days after seeding), cardiomyocytes were incubated with Fura-2AM (Invitrogen, Waltham, MA, USA) (10 µM) for 45 min at 37 °C and 5% CO_2_. Cells were then washed with phosphate-buffered saline (PBS) (three times) and incubated at 37 °C for a further 10 min in fully supplemented Claycomb medium to allow the dye to de-esterify. Cells were then mounted onto an inverted microscope connected to a video edge-recognition system (IonOptix, MyoCam-S, Dublin, Ireland) and optoscan monochromator fluorescence photometry (Cairn Research, Faversham, Kent, UK). Normal Ca^2+^ waves were recorded for a period of 1 min and calf thymus histones were then gently perfused across the cells and Ca^2+^ flux changes were recorded for 30 min. In experiments where the anti-histone single chain variable fragment (ahscFv) was used, this was pre-incubated with cells for 1 min prior to histone treatment. Fluorescence signals were elicited by alternate excitations at 340 and 380 nm wavelengths, at 250 Hz and recorded at 510 nm through a photomultiplier tube. To quantify [Ca^2+^]_i_, cells were perfused with 10 mM caffeine (Sigma-Aldrich, Gillingham, UK) to record the maximum (Rmax) and then with 25 mM EDTA to obtain the minimum (Rmin) intensities, as previously described [8,26]. All experiments were performed at 37 °C. Experiments were performed on spontaneously contracting as well as on paced cardiomyocytes (at 5 Hz) with comparable results.

### 4.4. Measurement of HL-1 Cardiomyocytes Contractility

Changes in HL-1 cardiomyocyte contractility was measured, as previously described [8,26]. Briefly, cells (10^6^ grown on 35 mm tissue culture dishes) were mounted onto an inverted microscope and the contraction (shortening) of cardiomyocytes was recorded using a video edge-recognition system (IonOptix, MyoCam-S, Dublin, Ireland). Only cells that showed a minimum of 0.5 µm shortening with a regular rhythm at the beginning of the recording were selected for experiments. After 1 min of baseline recording, histones were gently perfused across the cells and contractility was recorded for 30 min. In experiments where a chemical inhibitor (5 nM Go6976 and10 nM LY333531) was used, cells were pre-incubated with the inhibitor for 60 min (pre-incubation of 1 min in the case of ahscFv), as described previously [8]. Different parameters of contractility, i.e., peak shortening, time to peak (TTP) (correlates with systolic duration), time to 90% re-lengthening (tR_90_) (correlates with diastolic duration), maximum velocity of shortening (+dL/dt) (correlates with maximum rise in ventricular pressure) and re-lengthening (−dL/dt) (correlates with maximum drop in ventricular pressure) were analysed using Ion-Wizard 6.0 software (IonOptix, Westwood, MA, USA). All recordings were performed at 37 °C. Experiments were performed on spontaneously contracting as well as on paced cardiomyocytes (at 5 Hz) with comparable results.

### 4.5. Whole Cell Lysates Preparation of HL-1 and Mouse Cardiomyocytes

HL-1 cardiomyocytes were first washed with ice-cold PBS (three times) and then lysed with clear lysis buffer (1% SDS, 10% Glycerol, 120 mM Tris-HCL, 25 mM EDTA, protease inhibitor cocktail (Sigma-Aldrich, Gillingham, UK) 1:500 dilution, sodium orthovanadate 2 nM, pH 6.8). In the case of murine cardiomyocytes, murine LVs were first cut into 1–2 mm pieces and washed with ice-cold PBS (three times). These were then suspended in clear lysis buffer and thoroughly dispersed and lysed using a cell homogenizer (Ultra-Turrax T8, IKA Labortechnik, Staufen, Germany).

### 4.6. Subcellular Fractionation of HL-1 and Mouse Cardiomyocytes

Sub-cellular fractionation of HL-1 cells and murine LV cardiomyocytes into cytoplasmic and membrane fractions was performed as previously described [8]. HL-1 cardiomyocytes were washed with ice-cold PBS (three times) followed by the addition of homogenization buffer (25 mM Tris, 2 mM EDTA, 10% Glycerol). HL-1 cardiomyocytes were then shredded by passing through a 31-gauge needle (20–30 times). The nuclear fraction and any un-fractionated (intact) cells were removed by centrifugation at 1000× *g* for 5 min (at 4 °C). The supernatant, which contains the cytosolic and membrane fractions, was then centrifuged at 100,000× *g* for 45 min. The supernatant (cytosolic fraction) was transferred to a new tube and SDS was added (final concentration of 1%) whilst the pellet (membrane fraction) was lysed with clear lysis buffer. For murine cardiomyocytes, LVs were first cut into 1–2 mm pieces and washed with ice-cold PBS (three times). The pieces were then suspended in homogenization buffer and dispersed into a cellular suspension using a cell homogenizer (Ultra-Turrax T8, IKA Labortechnik, Staufen, Germany). Subcellular fractionation into cytoplasmic and membrane fractions was then performed as described above for HL-1 cardiomyocytes.

Myofilament fractionation of HL-1 cardiomyocytes and mouse LV cardiomyocytes was performed as previously described [8]. Briefly, HL-1 cardiomyocytes, were permeabilized by adding 1% triton X-100 (prepared in ice-cold PBS) on ice for 10 min. The triton-soluble fraction was transferred into a separate tube and SDS was added (final concentration of 1%). The remaining adherent fraction (triton-insoluble) (constituted mainly of the myofilaments) was lysed using clear lysis buffer. For murine cardiomyocytes, LV was first chopped and washed with PBS, as described above. The pieces were then suspended in 1% triton X-100 and dispersed using a cell homogenizer (Ultra-Turrax T8, IKA Labortechnik, Staufen, Germany). The suspension was left on ice for 10 min to permeabilize the cells and then centrifuged at 1000× *g* for 5 min. The supernatant (triton-soluble fraction containing both cytoplasmic and membrane compartments) was transferred into a new tube and SDS added (final concentration of 1%). The pellet (triton-insoluble which consists of the myofilament fraction) was washed with ice-cold PBS (three times) and then lysed using clear lysis buffer.

### 4.7. Western Blotting

All protein samples (whole cell lysates or from different sub-cellular fractions) were ultrasonicated and boiled at 100 °C for 10 min. Protein determination was performed using the Bio-Rad DC Protein Assay kit. Equal amounts of proteins (50 μg) were loaded into 10–15% SDS-PAGE gels and subjected to electrophoresis at 30 mAMP/gel for 60 min then transferred at 400 mAMP over 60 min to Immobilon-P PVDF transfer membranes. Membranes were then blocked with 5% milk in TBS-T for 60 min at room temperature. Primary antibodies against PKCα PKCβI, PKCβII, PKCε, PKCδ, PKCη (all from Santa Cruz Biotechnology, Wembley, UK), PKCζ, cTnI, cTnI (phosphor-T144), cTnI (phosphor-S43), pan-cadherin (all from Abcam, Cambridge, UK) and GAPDH (Cell Signalling, London, UK) were diluted in a concentration of 1:500–1:40,000 (according to the antibody) in 5% milk in TBS-T and incubated with the membrane for 12 h at 4 °C. The membrane was then washed for 15 min with TBS-T (three times) and then incubated with an anti-rabbit or anti-mouse (according to the primary antibody) HRP-labelled secondary antibody (Santa Cruz Biotechnology, Wembley, UK) in a concentration of 1:10,000 in 5% milk in TBS-T for 60 min. The membrane was then washed with TBS-T for 15 min (three times) and protein signals were assessed by chemiluminescent detection using the G box gel imaging system (Syngene, Cambridge, UK) and GeneSnap software version 7 (Syngene, Cambridge, UK).

### 4.8. Development of Anti-Histone Single Chain Variable Fragment Anti-Body (ahscFv)

Anti-histone single chain variable fragment (ahscFv) antibody was produced as previously described [22,26], based on sequences of complementarity determining regions (CDRs) of anti-histone antibodies from mice with autoimmune disorders [66] and was expressed in *Escherichia coli* and purified using his-binding resin. The histone-specific binding properties of ahscFv has been previously validated against non-specific antibodies [22].

### 4.9. Culture of Endothelial Cells

Immortalized EA.hy926 endothelial cells (ATCC, Virginia USA) were cultured as previously described [22] in Dulbecco’s modified Eagle’s Medium (DMEM, Sigma) supplemented with 10% foetal calf serum, 2 mM L-glutamine and 100 U/mL penicillin–streptomycin.

### 4.10. Statistics

Multiple group differences were assessed by analysis of variance test followed by Bonferroni’s correction. Statistical differences between two independent groups were tested using Student’s t test. *p* < 0.05 indicated a statistically significant difference. All statistical analyses were performed using SPSS software (version 22).

## 5. Conclusions

Extracellular histones at clinically relevant concentrations from our previous studies of sepsis induce cardiac depression. One potential mechanism is histone-induced Ca^2+^ influx that activated PKCα to phosphorylate cTnIS43 and T144. This work therefore suggests a role for the PKCα-cTnI pathway in mediating cardiomyocyte dysfunction associated with exposure to high levels of circulating histones in sepsis.

## Data Availability

The data presented in this study are available on reasonable request from the corresponding authors.
